# Relations between teacher–child interaction quality and children’s playfulness

**DOI:** 10.1080/03004430.2024.2356242

**Published:** 2024-05-22

**Authors:** Cornelia Rüdisüli, Isabelle Duss, Patricia Lannen, Corina Wustmann Seiler

**Affiliations:** a Department of Research and Development, Zurich University of Teacher Education, Zurich, Switzerland; b Marie Meierhofer Children’s Institute, University of Zurich, Zurich, Switzerland

**Keywords:** Children’s playfulness, teacher–child interaction quality, quality of children's play, kindergarten, childcare center

## Abstract

Adults’ behaviour in interactions with children is assumed to influence children’s playfulness. However, little is known about how the quality of teacher–child interaction in early childhood education and care affects the development of children’s playfulness, although the interaction quality has been identified as a strong predictor of children's development in various domains. The present study examined cross-sectional and longitudinal associations between children’s multidimensional playfulness and the quality of teacher–child interactions. At first measurement, 62 teachers in early childhood education and care (ECEC) were observed to assess the quality of their interactions with children using the standardized CLASS Toddler scale. The playfulness of 393 children was assessed using the children’s playfulness scale at the same time and one year later. No significant effects were found cross-sectionally. Longitudinally, high interaction quality in learning support was marginally negatively related to children’s total score of playfulness and significantly negatively to the playfulness dimensions of cognitive and physical spontaneity. We discuss which teaching styles might hamper or promote children’s playfulness.

## Introduction

Young children spend increasing time in institutions of early childhood education and care (ECEC), where they are engaged in play for much of the time (Wood, 2007). Through play, young children explore the world and acquire a wide range of competencies (Whitebread et al., 2017). The construct of playfulness describes the quality of children's play: their ability and willingness to play and their enjoyment of it (Barnett, 1991). Vygotsky’s social-constructivist theories imply the importance of teachers’ active role in creating such stimulating learning opportunities in interactions with young children (Wood & Bennett, 1998). Previous studies have shown that adults’ emotional availability and sensitivity in interactions can enhance children’s playfulness (Chiarello, Huntington, & Huntington, 2006; Wright, 2015). However, the broad dyadic construct of teacher–child interactions has not yet been examined as a predictor of children’s playfulness, even though interaction quality in ECEC has been identified as key to children’s engagement, effort, and other significant social-emotional and cognitive outcomes of children’s learning and development (e.g. Mashburn et al., 2008). In this study, we addressed this research gap longitudinally by analysing relationships between teacher–child interaction quality during a standardized observation at a first measurement (T1) and children’s playfulness assessed by teacher and parent ratings at the first (T1) and a second measurement (T2) one year apart.

### Children’s playfulness – the quality of children's play

Children’s playfulness is understood as the quality of children’s play and includes flexibility and enjoyment of play (Hamm, 2006). Children with high levels of playfulness tend to be characterized by spontaneity and adaptability, both in their play and in their social interactions (Cornelli Sanderson, 2010). Research about children’s playfulness addresses the child’s general approach to play and captures the quality of children’s play based on various play situations in their natural context (Bundy, 1997). Based on Cooper’s (2000) contextual model of play and playfulness, it is assumed that the child’s immediate environment including physical and social elements encourage or limits the quality of their play activity. Children’s playfulness is defined as a multidimensional construct that includes cognitive, social, emotional, and motor elements of play (Barnett, 1991). According to Lieberman (1977), five dimensions of playfulness can be distinguished: (1) cognitive spontaneity, (2) manifest joy, (3) physical spontaneity, (4) sense of humour, and (5) social spontaneity. Cognitive spontaneity reflects children’s imagination skills, such as inventing new games and assuming different roles in play. Manifest joy is shown in children’s engagement, enthusiasm, and emotions in play. Physical spontaneity refers to children’s coordination and joy of movement in their play. Sense of humour describes children’s joking, teasing, and clownish behaviour during play. And social spontaneity captures children’s interactions with others during play, including cooperating, sharing, and leading.

Research has shown that children’s playfulness is significantly related to child characteristics and skills such as active coping, emotion regulation, prosocial behaviour, and creativity (Ata & Macun, 2022; Ruckser-Scherb, 2010; Trevlas, Matsouka, & Zachopoulou, 2003). Furthermore, recent studies have demonstrated the role of children’s playfulness in predicting their future cognitive and social-emotional development (Fung & Chung, 2022; Waldman-Levi, Bundy, & Shai, 2022).

### The development of children’s playfulness

In general, playfulness is understood as the result of healthy development in the absence of physical or psychological trauma (Gordon, 2014). To date, however, we know little about how playfulness develops over time and what predicts its development. Children’s playfulness was initially defined as a behaviour trait of the individual child that remains constant over time (Barnett, 1991; Lieberman, 1977). In contrast, previous research has found positive correlations between age and children’s playfulness (Ata & Macun, 2022; Wustmann Seiler, Lannen, Duss, & Sticca, 2021) and that playfulness of toddlers increased over time (Waldman-Levi et al., 2022). This results in an understanding of playfulness as changeable, particularly in the early years of life (e.g. Reed, Dunbar, & Bundy, 2000). Clinical studies in occupational therapy have revealed small effects of interventions such as play groups and parent training (Bundy et al., 2011; Fabrizi, 2015; Okimoto, Bundy, & Hanzlik, 2000). Other studies have demonstrated that children’s playfulness differs in various settings and group constellations (Pinchover, Shulman, & Bundy, 2016; Rigby & Gaik, 2007). In addition, studies have shown that children’s playfulness relates to teachers’ and parents’ playfulness (Pinchover, 2017; Wustmann Seiler et al., 2021) and to support from caregivers in the child’s environment who provide safety and play opportunities (Bundy, Waugh, & Brentnall, 2009; Hamm, 2006). Moreover, the associations between children’s playfulness and the characteristics of the parents’ interactions, including their responsiveness and sensitivity (Chiarello et al., 2006; Wright, 2015), indicate the central role of adults in the development of children’s playfulness. However, to the best of our knowledge, only Pinchover and Shulman (2019) have previously examined the role of teachers’ interactions with children in children’s playfulness. These authors point to the importance of teacher–child interaction quality for children with autism spectrum disorders because high teacher emotional availability eliminated the negative association between problem behaviour and playfulness in these children.

### Teacher–child interaction quality

High-quality teacher–child interactions are characterized by sensitive and stimulating teaching that can be identified dynamically in children’s and teachers’ behaviour. Teacher–child interactions are considered a key element in supporting effective early learning and multiple aspects of child development in ECEC (e.g. Burchinal et al., 2008; Phillips & Lowenstein, 2011). They are viewed as essential proximal processes, sometimes measured by process quality, through which teachers can contribute directly to children’s learning and development (Pianta, Hamre, & Nguyen, 2020). Of all indicators of structure and experience in ECEC, the observed quality of teacher–child interactions accounts for the greatest variance in children’s current and later performance (Pianta & Hofkens, 2023). Previous studies have shown that high-quality teacher–child interactions play an important role in children’s development of academic skills such as language, science, and mathematics (e.g. Hu et al., 2020; Mashburn et al., 2008) and in the development of social-emotional skills such as prosocial behaviour, engagement, and self-regulation (e.g. Broekhuizen, Slot, van Aken, & Dubas, 2017; Castro, Granlund, & Almqvist, 2017). The Classroom Assessment Scoring System (CLASS), based on the Teaching Through Interactions (TTI) framework, is widely used to assess the quality of interactions between teachers and children (Pianta & Hofkens, 2023). It is a standardized and validated external observation tool and can be applied to various age groups. In the CLASS Toddler version for children aged 18–36 months, quality of interactions is measured in two domains: (1) emotional and behavioural support and (2) engaged support for learning. Emotional and behavioural support measures the emotional climate of the classroom, teachers’ sensitivity to children’s needs, and their guidance of children’s behaviour. Engaged support for learning assesses the degree to which teachers offer opportunities and feedback to enhance children’s thinking and language development (La Paro, Hamre, & Pianta, 2009). Various previous research has shown that teachers scored higher in emotional and behavioural support than in engaged support for learning (Burchinal et al., 2008; Perren, Frei, & Herrmann, 2016). In addition, teachers’ emotional and behavioural support tends to predict social-emotional outcomes, whereas teachers’ engaged support for learning tends to predict academic and cognitive outcomes (e.g. Castro et al., 2017; Mashburn et al., 2008). Various findings indicate that the effects of interactions in ECEC also depend on children’s individual and familial characteristics, such as their abilities and the socio-economic background of their families (Coelho, Cadima, & Pinto, 2019; Peisner-Feinberg et al., 2001). However, recent research demonstrated that even after controlling for multiple child and familial characteristics, the quality of teacher–child interactions remains a strong predictor of children’s outcomes (e.g. Pinto et al., 2019; Salminen, Pakarinen, Poikkeus, Laakso, & Lerkkanen, 2022).

### Summary and present study

Play is considered the main learning activity of children in ECEC, and its quality can be described by the construct of playfulness. Research indicates that adults can stimulate children's playfulness through responsive and sensitive behaviour in interactions with them (Chiarello et al., 2006). However, these findings mainly relate to parental behaviour or to a clinical context and focus on the sensitivity of the behaviour. No studies have been identified that examine teacher–child interactions as a dyadic system and include teachers’ learning support as well as emotional elements of the interaction. Previous studies have confirmed the significant role of teacher–child interaction quality in ECEC in children’s academic and social-emotional outcomes (e.g. Pinto et al., 2019). Therefore, high-quality teacher–child interactions can also be assumed to promote the development of children's playfulness, here assessed as a multidimensional construct including cognitive, social, emotional, and motor elements. The present study is the first to examine the quality of teacher–child interactions as a predictor of children’s playfulness cross-sectionally and longitudinally while controlling for children’s age and gender.

## Materials and methods

### Procedure

The study is part of the longitudinal study titled ‘Playfulness in Early Childhood (Playful)’. Participants were recruited through ECEC institutions with a broad call for applications via channels such as newsletters, school administrations, and organizations for ECEC. In selecting the sample, we considered urban and rural areas, various cantons, and various pedagogical settings. At the first measurement (T1) in spring 2021, we observed the quality of teacher–child interactions in childcare centres and kindergartens. At the same time, children’s playfulness was assessed with an online questionnaire to parents and teachers. At the second measurement (T2), one year later, parents and teachers again rated children’s playfulness with the same questionnaire. The present analyses include only those children who stayed with the same teacher throughout the study and from whom we received information on their playfulness at both measurements. The questionnaires were administered online with Survalyzer software.

The Ethics Committee of the Faculty of Philosophy at the University of Zurich, Switzerland, reviewed and approved the study (ethics approval number 20.12.13). A written study description was provided to all teachers and parents to inform them about the objectives and procedures of the study. They were also informed of their right to withdraw from participation at any point without providing a reason. Additionally, their data would be collected anonymously and used only for scientific research purposes. Prior to their involvement, all participants provided their written informed consent.

### Participants

The initial sample at T1 consisted of 62 ECEC teachers in childcare centres (*n* = 25) and kindergartens (*n* = 37). Childcare centres are private institutions for children from infancy onwards. Kindergarten is part of the public school system; it is obligatory for children from the age of four years and usually lasts two years. Kindergarten teachers typically hold bachelor’s degrees, and teachers at childcare centres have completed vocational training. At kindergarten, children usually spend five mornings a week for four hours without lunch break. In childcare centres, the children all usually stay for a full day, but the number of days varies from one to five days per week. Even though childcare centres and kindergartens differ from each other in funding, age range, and staff training, free play in both is considered a central part of the early education curriculum (e.g. EDK, 2016). ECEC settings in the present sample were located in the German-speaking part of Switzerland. The ages of the 62 participating teachers ranged from 23 to 63 years (*M* = 38.2, *SD* = 11.7), and their work experience varied from 1 to 37 years (*M* = 13.8, *SD* = 9.9). On average, teachers worked 35 hours a week with a range from 19 to 42 hours. Most of the teacher sample were female (95.1%) and had a Swiss background (90.2%).

A total of 393 children between 2 and 6 years at T1 (*M*_age_ = 4.5; *SD*_age_ = 1.3; 44.0% female) participated in the study at both measurements. Of these, 44.8% attended childcare centres and 55.2% kindergartens at T1. The number of participating children in each ECEC group varied between 2 and 15 (*M* = 6.3, *SD* = 2.8). On average, the children attended childcare centres 2.4 days a week (*SD* = 1.12) and kindergarten 2.6 days a week (*SD* = 0.3). However, the time that children spend in kindergarten is generally spread over 5 days, whereas children stay at the daycare centre for fewer days but for longer hours. Most children were of Swiss origin (82.8%) and German-speaking (local language at home; 88.5%). Some 49.8% of their mothers had a university degree, and 41.3% had completed vocational training.

### Measures

#### Teacher–child interaction quality

The quality of interactions between teacher and children was assessed using the Classroom Assessment Scoring System – Toddler version (CLASS Toddler; La Paro et al., 2009), a standardized observation instrument that is widely used internationally. It was originally developed in the USA for children aged 18–36 months and focuses on unstructured time. The CLASS version Pre-K (Pianta, La Paro, & Hamre, 2008) was developed for older children, with whom more instructional activities take place in preschool. In the Swiss ECEC settings, children are granted more unstructured time, such as free play, than children of the same age in the USA (Coelho, et al., 2021). Therefore, CLASS Toddler is also used with older children in Swiss ECEC settings (e.g. Diebold & Perren, 2022). In addition, the same CLASS version could be used for both ECEC settings in our sample, childcare centres and kindergarten.

The CLASS Toddler scale consists of eight dimensions: (1) positive climate, (2) negative climate, (3) teacher sensitivity, (4) regard for child perspectives, (5) behavioural guidance, (6) facilitation of learning and development, (7) quality of feedback, and (8) language modelling. The first five dimensions can be assigned to the domain of emotional and behavioural support and the final three dimensions to engaged support for learning. The two-dimensional structure of the CLASS Toddler has been statistically confirmed in various studies as well in the Swiss context, where the dimension of negative climate was often excluded due to ceiling effects or low variance (e.g. Diebold & Perren, 2022; Pakarinen et al., 2010)

In the present study, four to six live observation cycles (*n* = 325, *M* = 5.24, *SD* = 0.61) of 15–20 min were conducted per classroom. The observations focused on the interactions between the main teacher and the children. The rating was done on site with a seven-point scale (1 = low quality, 7 = high quality) directly after each observation cycle. Based on these observation cycles, a mean value per teacher was calculated for each dimension. Due to ceiling effects, the negative climate dimension (*M* = 6.86, *SD *= 0.19) was also excluded. Both emotional and behavioural support (Cronbach’s *α* = 0.89) and engaged support for learning (Cronbach’s *α* = 0.80) had satisfactory internal consistency. Researchers rating the data completed a two-day training course and certification for use of the instrument. Upon successful certification, researchers passed a reliability test by achieving at least 80% agreement with a master coder. Nevertheless, in the present study, double rating with two parallel raters was performed for 19% of all cycles to demonstrate interrater reliability for the Swiss context. Interrater agreement was satisfactory except for the quality of feedback dimension, with a value just below the limit (ICC = .58); for all other dimensions, interrater agreement had ICC values between .69 and .84.

#### Children’s playfulness

Children’s playfulness was assessed using the German version of the Children’s Playfulness Scale (CPS; Barnett, 1991; Wustmann Seiler et al., 2021). The CPS consists of five dimensions: (1) manifest joy, (2) cognitive spontaneity, (3) social spontaneity, (4) sense of humour, and (5) physical spontaneity. The scale comprises a total of 23 items that are rated on a 5-point Likert scale (1 = doesn’t sound at all like the child; 5 = sounds exactly like the child). In the present study, we assessed children’s playfulness using parent and teacher reports, as children's play behaviour can vary depending on the environments and play partners. By averaging the ratings of parents and teachers, the global playfulness of each individual child can be captured more validly, and parent and teacher biases, that may arise from only witnessing the child in one context, can be minimized (Rentzou, 2013). Confirmatory factor analyses were carried out to verify the factor structure of the five dimensions at first order and of the total score at second order (McDonald’s Omega ΩT1 = .86; ΩT2 = .88). The models showed acceptable fits (based on Hu & Bentler, 1999), with two error correlations at T1 and one error correlation at T2 (see Table 1). For each of the five playfulness dimensions, the items with the best factor loadings and reliability values were selected. The dimension of manifest joy contained four items, for example ‘the child shows enthusiasm during play’ (McDonald’s Omega ΩT1 = .70; ΩT2 =  .83). The cognitive spontaneity dimension included three items, for example ‘the child invents his/her own games to play’ (McDonald’s Omega ΩT1 = .65; ΩT2 = .74). Three items were selected for the dimension of social spontaneity, for example ‘the child plays cooperatively with other children’ (McDonald’s Omega ΩT1 = .81; ΩT2 = .83). The sense of humour dimension contained four items, for example ‘the child enjoys joking with other children’ (McDonald’s Omega ΩT1 = .82; ΩT2 = .85). And finally, three items comprised the physical spontaneity dimension, for example ‘the child runs (skips, hops, jumps) a lot in play’ (McDonald’s Omega ΩT1 = .85; ΩT2 = .87).

**Table 1.** Model fit indices of the confirmatory factor analysis of children’s playfulness.

	*X^2^*	*df*	*p*	CFI	RMSEA	SRMR
First order T1	273.81	107	0.000	0.924	0.063	0.052
Second order T1	303.84	112	0.000	0.913	0.066	0.062
First order T2	243.02	108	0.000	0.945	0.056	0.046
Second order T2	286.09	113	0.000	0.930	0.062	0.060

Note: *χ*^*2* ^= Satorra-Bentler scaled Chi-square; *df* = degrees of freedom; *p* = probability of type I error; CFI = Comparative Fit Index; RMSEA = Root Mean Square Error of Approximation; SRMR = Standardized Root Mean Square Residual.

Measurement invariance was tested by comparing different invariance models up to a scalar invariance for the first order model and up to partial scalar invariance for the second order model with one intercept at second order (sense of humour at T1) constrained to equality (see Table 2). The findings indicated a stability over time of the latent multidimensional construct of children’s playfulness represented across the selected items (first and second order).

**Table 2.** Model fit comparison of the playfulness scores for the examination of measurement invariance.

	*χ^2^*	*df*	*p*	CFI	RMSEA	SRMR	Δχ2	Δdf	*p*
**Dimensions of Playfulness at first order**									
Configural	785.90	462	0.000	0.943	0.042	0.049			
Metric	803.54	474	0.000	0.942	0.042	0.053	17.644	12	0.123
Scalar	815.81	486	0.000	0.942	0.042	0.055	12.267	12	0.413
**Total Score of Playfulness at second order**									
Configural	873.64	491	0.000	0.933	0.045	0.064			
Metric	888.84	507	0.000	0.933	0.044	0.068	15.202	16	0.416
Scalar	936.46	523	0.000	0.927	0.045	0.071	47.623	16	0.000
Partial scalar	909.18	522	0.000	0.932	0.043	0.071	20.345	15	0.148

Note. *χ*^*2* ^= Satorra-Bentler scaled Chi-square; *df* = degrees of freedom; *p* = probability of type I error; CFI = Comparative Fit Index; RMSEA = Root Mean Square Error of Approximation; SRMR = Standardized Root Mean Square Residual; Δ = Difference Value.

### Data analysis

First, we computed descriptive statistics and bivariate correlations of all study variables. Second, structural equation models were constructed to predict children’s playfulness from the two domains of teacher–child interaction quality cross-sectionally and longitudinally, separately for the five dimensions of playfulness at first order and for the total score of playfulness at second order. The two domains of interaction quality were analysed in distinct models because of their high correlation (*r* = .73, *p* < .001).

For cross-sectional analysis, children’s playfulness at T1 was entered as a dependent latent variable at first and second order, both domains of teacher–child interaction quality were entered separately as manifest predictors, and age and gender were entered as control variables due to their predictive significance in previous studies (e.g. Barnett, 1991; Keleş & Yurt, 2017). In the longitudinal analysis, the same setup was modelled, but in addition, children's playfulness at T1 was entered latently as a third control variable. All predictors were allowed to correlate with each other. The model fit of all models were acceptable based on Hu and Bentler (1999): first order, *X^2^*(578) = 1041.043–1043.068, *p* = 0.000, CFI = 0.922, RMSEA = 0.045, SRMR = 0.058; second order, *X^2^*(618) = 1203.205−1205.256, *p* = 0.000, CFI = 0.900, RMSEA = 0.049, SRMR = 0.073. We used the Huber-White sandwich estimator (Freedman, 2006) to analyse the hierarchical data structure, which involved children nested within ECEC groups (ICC of item level: 0.04–0.22). The full information maximum likelihood method (FIML) was used to address missing data. All analyses were conducted using MPLUS version 8.8 (Muthén & Muthén, 1998-2018).

## Results

### Descriptive statistics

Table 3 presents mean scores and bivariate correlations of all study variables at T1 and T2. Teachers had higher scores in the CLASS Toddler domain of emotional and behavioural support than in engaged support for learning. Both domains were significantly and highly positively intercorrelated. Regarding children’s playfulness, at both measurements, the manifest joy dimension achieved the highest mean values, followed by the dimensions of physical spontaneity, cognitive spontaneity, social spontaneity, and sense of humour with the lowest mean values. In addition, most dimensions were correlated significantly and positively with each other at low to high levels.

**Table 3.** Descriptive statistics and bivariate correlations of all study variables.

		*N*	*M*	*SD*	1	2	3	4	5	6	7	8	9	10	11	12	13	14	15
**Playfulness scores at T1**																			
1	TPF T1	393	4.24	0.38	1														
2	MJ T1	393	4.24	0.40	–	1													
3	CS T1	393	3.91	0.56	–	.89***	1												
4	SS T1	393	3.73	0.61	–	.42***	.47***	1											
5	SH T1	393	3.03	0.51	–	.66***	.72***	.41***	1										
6	PS T1	393	4.03	0.64	–	.61***	.39***	.14*	.52***	1									
**Playfulness scores at T2**																			
7	TPF T2	393	4.33	0.44	.60***	–	–	–	–	–	1								
8	MJ T2	393	4.33	0.46	–	.56***	.47***	.23**	.46***	.39***	–	1							
9	CS T2	393	4.04	0.69	–	.53***	.61***	.22**	.40***	.23***	–	.85***	1						
10	SS T2	393	3.90	0.65	–	.17*	.21*	.64***	.22**	.07	–	.43***	.45***	1					
11	SH T2	393	3.28	0.56	–	.46***	.42***	.20**	.63***	.40***	–	.79***	.68***	.38***	1				
12	PS T2	393	4.15	0.68	–	.40***	.23***	.09	.47***	.66***	–	.68***	.38***	.17*	.65***	1			
**Teacher—child interaction quality**																			
13	EBS	393	5.43	0.77	.02	−.01	−.03	−.03	.08	.06	−.01	.00	−.07	−.04	.02	.01	1		
14	ESL	393	3.37	0.82	.04	.01	.02	−.01	.06	.03	−.06	−.03	−.14*	−.09	−.03	−.05	.73***	1	
**Control variables**																			
15	Gender^a^	393	0.44	–	.08	.07	.18*	.17***	.02	−.12*	.08	.11	.23***	.09	−.02	−.17**	.07	−.01	1
16	Age^b^	393	54.21	15.31	.26***	.13*	.34***	.25***	.37***	.01	.18*	.15*	.15	.12	.23***	.14*	−.02	.12	.01

Note: TPF = total score of playfulness, MJ = manifest joy, CS = cognitive spontaneity, SS = social spontaneity, SH = sense of humour, PS = physical spontaneity. EBS = emotional and behavioural support, ESL = engaged support for learning.

All playfulness scores were modelled as a latent variable. ^a^0 = male; 1 = female; ^b^ in months.

*p < .050; **p < .010; ***p < .001.

Bivariate correlations between the two domains of teacher–child interaction quality and different scores of children’s playfulness at T1 and T2 indicated a low yet significant negative correlation between the domain of engaged support for learning and the cognitive spontaneity dimension at T2. No other playfulness scores were significantly correlated with either domain of teacher–child interaction quality.

### Cross-sectional relations between children’s playfulness and teacher–child interaction quality

Standardized results of the models for the prediction of children’s playfulness at T1 and T2 are reported in Tables 4 and 5. Neither domain of teacher–child interaction quality significantly predicted the total score of playfulness cross-sectionally. No significant predictive effects of teacher–child interaction quality emerged for any of the five dimensions of playfulness in the cross-section either.

**Table 4.** Cross-sectional and longitudinal effects of emotional and behavioural support on children’s playfulness.

	Cross section						Longitudinal					
	TPF T1 β *(SE)*	MJ T1 β *(SE)*	CS T1 β *(SE)*	SS T1 β *(SE)*	SH T1 β *(SE)*	PS T1 β *(SE)*	TPF T2 β *(SE)*	MJ T2 β *(SE)*	CS T2 β *(SE)*	SS T2 β *(SE)*	SH T2 β *(SE)*	PS T2 β *(SE)*
Age^a^	.26 (0.07)***	.13 (0.06)*	.34 (0.07)***	.24 (0.06)***	.37 (0.06)***	.01 (0.06)	.02 (0.06)	.08 (0.06)	-.07 (0.07)	-.05 (0.06)	.00 (0.06)	.13 (0.05)**
Gender^b^	.09 (0.05)	.07 (0.06)	.18 (0.07)**	.17 (0.05)***	.01 (0.05)	−.12 (0.05)*	.03 (0.05)	.07 (0.05)	.11 (0.07)^†^	−.02 (0.05)	−.03 (0.05)	−.10 (0.05)^†^
PF T1	–	–	–	–	–	–	.59 (0.05)***	.55 (0.05)***	.62 (0.05)***	.66 (0.04)***	.62 (0.04)***	.63 (0.05)***
EBS	.02 (0.08)	−.01 (0.08)	−.04 (0.10)	−.03 (0.07)	.08 (0.07)	.07 (0.07)	−.03 (0.05)	.00 (0.07)	−.06 (0.06)	−.02 (0.06)	−.03 (0.05)	−.02 (0.06)
*R^2^*	.08 (0.04)^†^	.02 (0.02)	.15 (0.05)**	.09 (0.03)**	.14 (0.05)**	.02 (0.02)	.36 (0.06)***	.33 (0.05)***	.40 (0.05)***	.42 (0.05)***	.39 (0.05)***	.43 (0.05)***

Note. TPF = total score of playfulness, MJ = manifest joy, CS = cognitive spontaneity, SS = social spontaneity, SH = sense of humour, PS = physical spontaneity. *β* = Standardized regression coefficients, SE = standard errors. PF = playfulness scores; EBS = emotional and behavioural support, ESL = engaged support for learning.

^a^ in months; ^b^0 = male; 1 = female.

^†^ *p* < 0.100; *p < .050; **p < .010; ***p < .001.

**Table 5.** Cross-sectional and longitudinal effects of engaged support for learning on children’s playfulness

	Cross section						Longitudinal					
	TPF T1 β *(SE)*	MJ T1 β *(SE)*	CS T1 β *(SE)*	SS T1 β *(SE)*	SH T1 β *(SE)*	PS T1 β *(SE)*	TPF T2 β *(SE)*	MJ T2 β *(SE)*	CS T2 β *(SE)*	SS T2 β *(SE)*	SH T2 β *(SE)*	PS T2 β *(SE)*
Age^a^	.26 (0.07)***	.13 (0.06)*	.34 (0.07)***	.25 (0.06)***	.37 (0.06)***	.01 (0.07)	.03 (0.06)	.08 (0.06)	-.05 (0.07)	-.04 (0.06)	.01 (0.06)	.14 (0.05)**
Gender^b^	.09 (0.05)	.07 (0.06)	.18 (0.07)**	.17 (0.05)***	.02 (0.05)	−.12 (0.05)*	.03 (0.05)	.07 (0.05)	.11 (0.06)^†^	−.02 (0.05)	−.03 (0.05)	−.10 (0.05)*
PF T1	–	–	–	–	–	–	.59 (0.06)***	.55 (0.05)***	.62 (0.05)***	.66 (0.04)***	.62 (0.05)***	.63 (0.05)***
ESL	.01 (0.07)	−.00 (0.07)	−.02 (0.08)	−.04 (0.07)	.01 (0.07)	.03 (0.08)	−.09 (0.06) ^†^	−.04 (0.07)	−.14 (0.05)**	−.07 (0.06)	−.07 (0.05)	−.09 (0.05)*
*R^2^*	.08 (0.04)^†^	.02 (0.02)	.15 (0.05)**	.09 (0.03)**	.14 (0.04)**	.02 (0.01)	.36 (0.06)***	.33 (0.05)***	.42 (0.06)***	.43 (0.05)***	.40 (0.05)***	.44 (0.06)***

Note. TPF = total score of playfulness, MJ = manifest joy, CS = cognitive spontaneity, SS = social spontaneity, SH = sense of humour, PS = physical spontaneity. *β* = Standardized regression coefficients, SE = standard errors. PF = playfulness scores; EBS = emotional and behavioural support, ESL = engaged support for learning.

^a^ in months; ^b^0 = male; 1 = female.

^†^ *p* < 0.100; *p < .050; **p < .010; ***p < .001.

### Longitudinal relations between children’s playfulness and teacher–child interaction quality

As presented in Tables 4 and 5, engaged support for learning had a negative, small but marginally significant effect for the long-term prediction of the total score of playfulness at T2, but the effect of emotional and behavioural support was not significant. Engaged support for learning had negative, small but significant effects on cognitive spontaneity and physical spontaneity. In other words, higher scores on engaged support of learning at T1 were linked to lower scores on these dimensions of children’s playfulness at T2. The negative effects on the other three dimensions, social spontaneity, manifest joy, and sense of humour, were low and not significant. The other domain of teacher–child interaction quality, emotional and behavioural support, showed no significant effect on any dimension of children’s playfulness in the long term.

### Additional results

Children’s age was significantly and positively related to the total score of playfulness at T1 at a low level, but gender did not have a noticeable effect. At T2, none of the control variables had a significant effect on the total score of playfulness. Children’s age significantly and positively predicted at T1 the dimensions of cognitive spontaneity, social spontaneity, and sense of humour at small to medium levels. However, children’s age showed no effect on the dimensions of manifest joy and physical spontaneity. In contrast, there was a small significant age effect at T2 on physical spontaneity, but not on other playfulness dimensions. At T1, girls were rated higher in cognitive and social spontaneity, whereas boys were rated higher in physical spontaneity. At T2, the gender effect was only significant in the physical spontaneity dimension. All the playfulness scores at T1 significantly positively predicted the scores of playfulness at T2 at high levels.

## Discussion

The aim of the present study was to investigate cross-sectional and longitudinal associations between children’s playfulness and the quality of teacher–child interactions in ECEC. We observed teacher–child interaction quality with both domains of the standardized CLASS Toddler scale: emotional and behavioural support and engaged support for learning. We also assessed children’s playfulness multidimensionally with a multi-informant approach twice at one-year intervals. Positive relations were expected, because the construct of playfulness reflects a dynamic view of children’s play and learning and includes cognitive and social-emotional elements predicted by high-quality teacher–child interaction in previous studies. Our results demonstrated no significant relation in the cross-section, but longitudinally significant negative effects of engaged support for learning on two dimensions of children’s playfulness, cognitive and physical spontaneity. These results are contrary to expectations and contribute to the discussion of the role of children’s playfulness in the pedagogical context.

### The construct of children’s playfulness

Children’s playfulness was assessed at two measurement points and modelled as latent factors with evidence of measurement invariance over time. All playfulness scores at T1 related significantly and strongly to the scores at T2 (*β* = .55–.66, *p* < .001) and thus showed stability over a period of one year. In addition, the mean value of the playfulness scores ranged between 3.03 and 4.33 on a 5-point Likert scale, the middle to upper range, and thus showed neither ceiling effects nor wide variance. As in previous studies, the manifest joy dimension was rated highest and the sense of humour dimension lowest (e.g. Fung & Chung, 2022). In line with previous studies (e.g. Keleş & Yurt, 2017), girls were rated lower in physical spontaneity and higher in social and cognitive spontaneity. In addition, age predicted children’s total score of playfulness only at T1.

### External assessment of teacher–child interaction quality in ECEC

The two-domain structure of the CLASS Toddler Scale, developed in the USA, was confirmed for the present Swiss sample and showed high internal consistency. Despite cultural differences in ECEC, such as fewer instructional activities in Switzerland than in the USA (Coelho et al., 2021), the mean values of the assessments in the two domains were comparable to previous studies in that emotional and behavioural support was rated higher than engaged support for learning (Burchinal et al., 2008).

### Effects of teacher—child interaction quality on children’s playfulness

The two domains of teacher–child interaction quality, emotional and behavioural support and engaged support of learning, were analysed separately. The results of the structural equation models showed no significant associations between the emotional and behavioural support domain and the playfulness scores cross-sectionally and longitudinally. This finding is contrary to expectations, as this domain encompasses elements such as a positive teacher–child relationship or teachers’ sensitivity. Previous studies have demonstrated a positive association between children’s playfulness and the quality of parental interactions, including responsiveness and sensitivity (Chiarello et al., 2006; Wright, 2015).

The engaged support of learning domain neither predicted children’s playfulness cross-sectionally. Longitudinally, however, the results revealed significant negative effects on the total score of children’s playfulness and the two dimensions of cognitive spontaneity and physical spontaneity, however on the total score only marginally significant. This suggests that when teachers facilitate activities to support children’s learning and expand their participation by providing feedback, they will likely improve academic and cognitive outcomes of young children (e.g. Mashburn et al., 2008; Salminen, Guedes, Lerkkanen, Pakarinen, & Cadima, 2021), but may inhibit the development of children's playfulness in the long term. The longitudinal effects indicate that teachers’ focus on support of learning may reflect a pedagogical attitude that manifests itself over time. This attitude could be characterized by interactions that require a high quality of feedback and stimulation of children’s thinking and language; this is accompanied by teachers’ structured and goal-orientated behaviour, which leaves little room for children’s spontaneity, creativity, and own ideas.

### Strengths and limitations

To the best of our knowledge, this is the first study to investigate the relationship between the quality of teacher–child interaction and the development of children’s playfulness. The CLASS Toddler scale, an internationally established, standardized, and validated instrument, was used to observe the quality of interactions in ECEC. In addition, children’s playfulness was recorded longitudinally at two measurements that used a multi-informant approach by including both parent and teacher reports. However, the present results must be interpreted with the following limitations: Even though the sample size of 393 children at both measurements exceeds the sample sizes of previous longitudinal studies on children’s playfulness (Waldman-Levi et al., 2022), the sample of 62 teachers as a reference for the predictor variables of interaction quality was rather small. The limited sample could be an explanation for the barely significant results. Nevertheless, the present study found zero or very small effects of teacher–child interaction quality on children’s playfulness, which is in line with findings in previous studies (Hong et al., 2019). The limited effects may be linked to the already high initial level of children’s playfulness or may indicate stronger predictors outside the institutional context.

In the present study, the frequency of the children’s ECEC attendance was not included as a control variable, due to the small variance and the different care structures at childcare centres and kindergartens. For instance, children in kindergartens are required by law to attend at least five mornings a week, whereas attendance at childcare centres can be determined by the parents and tends to vary. Nevertheless, children who spend more time in ECEC can be assumed to be more affected by the quality of teacher–child interactions. In addition, specific characteristics of the two ECEC settings childcare centre and kindergarten (e.g. teachers’ educational background) should be investigated in a larger sample of teachers.

The quality of teacher–child interaction can also be assumed to vary with the number of adults present and with consequent changes in the teacher–child ratio (Smidt & Embacher, 2023; Thomason & La Paro, 2009). However, observations in the present study focused only on the main teachers, who spend most time with the children and are responsible for the group. Including a second measurement of teacher–child interaction quality would allow both a second cross-sectional analysis at T2 and a longitudinal analysis. Moreover, the CLASS ratings in this study were conducted in diverse activities in ECEC, such as during routines, guided circle time, and free play. Further research on children’s playfulness might usefully focus solely on interactions during free play and adapt the observation scale by observing the teachers’ role during free play. However, it is still unclear whether the CLASS Toddler scale is suitable for assessing teacher–child interactions during free play, even if this CLASS version is more appropriate than the Pre-K version (Pianta et al., 2008) for older children, which focuses more on instructional support (Diebold & Perren, 2022). Especially because previous studies have shown that the quality of interactions during free play is generally lower than during guided activities (Perren et al., 2016), particularly in countries with established curriculum guidelines in ECEC such as Switzerland (Cadima et al., 2023), the question arises what other aspects or domains of interaction are relevant to supporting children in their free play.

### Conclusion

The construct of children’s playfulness describes the quality of children’s play and is an expression of their well-being and health (e.g. Youell, 2008). Children’s playfulness is assumed to be related to environmental factors such as social interactions. The present study showed that the quality of teacher–child interaction affected children’s playfulness only marginally. However, when teachers focus on supporting learning, they may inhibit the development of children’s playfulness in the long term. This can be explained by interactions during which teachers may encourage children’s development of thinking and learning through a goal-oriented approach, which perhaps unintentionally overlooks the inherent spontaneity of their play. The question arises whether more child-centred interactions that focus less on learning outcomes and more on children’s free play could be more beneficial for the development of children’s playfulness in ECEC.

## Data Availability

The datasets generated for this study are available on request from the corresponding author.

## References

[CIT0001] Ata, S., & Macun, B. (2022). Play tendency and prosociality in early childhood. *Early Child Development and Care*, *192*(13), 2087–2098. doi:10.1080/03004430.2021.1988943

[CIT0002] Barnett, L. (1991). The playful child: Measurement of a disposition to play. *Play & Culture*, *4*, 51–74.

[CIT0003] Broekhuizen, M. L., Slot, P. L., van Aken, M. A., & Dubas, J. S. (2017). Teachers’ emotional and behavioral support and preschoolers’ self-regulation: Relations with social and emotional skills during play. *Early Education and Development*, *28*(2), 135–153. doi:10.1080/10409289.2016.1206458

[CIT0004] Bundy, A. C. (1997). Play and playfulness: What to look for. In D. Parham, & L. Fazio (Eds.), *Play in occupational therapy for children* (pp. 52–66). St. Louis: Mosby.

[CIT0005] Bundy, A. C., Kolrosova, J., Paguinto, S. G., Bray, P., Swain, B., Wallen, M., … Engelen, L. (2011). Comparing the effectiveness of a parent group intervention with child-based intervention for promoting playfulness. *The Israeli Journal of Occupational Therapy*, *20*(4), 95–113.

[CIT0006] Bundy, A. C., Waugh, K., & Brentnall, J. (2009). Developing assessments that account for the role of the environment: An example using the test of playfulness and test of environmental supportiveness. *OTJR: Occupation, Participation and Health*, *29*(3), 135–143. doi:10.3928/15394492-20090611-06

[CIT0007] Burchinal, M., Howes, C., Pianta, R., Bryant, D., Early, D., Clifford, R., & Barbarin, O. (2008). Predicting child outcomes at the end of kindergarten from the quality of pre-kindergarten teacher–child interactions and instruction. *Applied Developmental Science*, *12*(3), 140–153. doi:10.1080/10888690802199418

[CIT0008] Cadima, J., Aguiar, C., Guedes, C., Wysłowska, O., Salminen, J., Slot, P., … Lerkkanen, M. K. (2023). Process quality in toddler classrooms in four European countries. *Early Education and Development*, *34*(7), 1565–1589. doi:10.1080/10409289.2022.2139548

[CIT0009] Castro, S., Granlund, M., & Almqvist, L. (2017). The relationship between classroom quality-related variables and engagement levels in Swedish preschool classrooms: A longitudinal study. *European Early Childhood Education Research Journal*, *25*(1), 122–135. doi:10.1080/1350293X.2015.1102413

[CIT0010] Chiarello, L. A., Huntington, A., & Huntington, A. (2006). A comparison of motor behaviors, interaction, and playfulness during mother-child and father-child play with children with motor delay. *Physical & Occupational Therapy in Pediatrics*, *26*(1-2), 129–151. doi:10.1300/J006v26n01_09 16938829

[CIT0011] Coelho, V., Åström, F., Nesbitt, K., Sjöman, M., Farran, D., Björck-Åkesson, E., … Pinto, A. I. (2021). Preschool practices in Sweden, Portugal, and the United States. *Early Childhood Research Quarterly*, *55*, 79–96. doi:10.1016/j.ecresq.2020.11.004

[CIT0012] Coelho, V., Cadima, J., & Pinto, A. I. (2019). Child engagement in inclusive preschools: Contributions of classroom quality and activity setting. *Early Education and Development*, *30*(6), 800–816. doi:10.1080/10409289.2019.1591046

[CIT0013] Cooper, R. J. (2000). The impact of child abuse on children’s play: A conceptual model. *Occupational Therapy International**,* *7*, 259-276. doi:10.1002/oti.127

[CIT0014] Cornelli Sanderson, R. (2010). Towards a new measure of playfulness: The capacity to fully and freely engage in play (Doctoral dissertation). Loyola University Chicago.

[CIT0015] Diebold, T., & Perren, S. (2022). Toddlers’ peer engagement in Swiss childcare: Contribution of individual and contextual characteristics. *European Journal of Psychology of Education*, *37*(3), 627–648. doi:10.1007/s10212-021-00552-2

[CIT0016] EDK (Deutschschweizer Erziehungsdirektoren-Konferenz) (2016). Lehrplan 21. [curriculum of schools in Switzerland]. https://www.lehrplan21.ch

[CIT0017] Fabrizi, S. E. (2015). Splashing our way to playfulness! An aquatic playgroup for young children with autism, a repeated measures design. *Journal of Occupational Therapy, Schools, & Early Intervention*, *8*(4), 292–306. doi:10.1080/19411243.2015.1116963

[CIT0018] Freedman, D. A. (2006). On the so-called “Huber sandwich estimator” and “robust standard errors”. *The American Statistician*, *60*(4), 299–302. doi:10.1198/000313006X152207

[CIT0019] Fung, W. K., and Chung, K. K. H. (2022). Parental play supportiveness and kindergartners’ peer problems: Children’s playfulness as a potential mediator. *Social Development*, *31*, 1126–1137. doi:10.1111/sode.12603

[CIT0020] Gordon, G. (2014). Well played: The origins and future of playfulness. *American Journal of Play*, *6*(2), 234–266.

[CIT0021] Hamm, E. M. (2006). Playfulness and the environmental support of play in children with and without developmental disabilities. *OTJR: Occupation, Participation and Health*, *26*(3), 88–96. doi:10.1177/153944920602600302

[CIT0022] Hong, S. L. S., Sabol, T. J., Burchinal, M. R., Tarullo, L., Zaslow, M., & Peisner-Feinberg, E. S. (2019). ECE quality indicators and child outcomes: Analyses of six large child care studies. *Early Childhood Research Quarterly**,* *49*, 202-217. doi:10.1016/j.ecresq.2019.06.009

[CIT0023] Hu, B. Y., Fan, X., Wu, Y., LoCasale-Crouch, J., & Song, Z. (2020). Teacher–child interaction quality and Chinese children’s academic and cognitive development: New perspectives from piecewise growth modeling. *Early Childhood Research Quarterly*, *51*, 242–255. doi:10.1016/j.ecresq.2019.10.003

[CIT0024] Hu, L. T., & Bentler, P. M. (1999). Cutoff criteria for fit indexes in covariance structure analysis: Conventional criteria versus new alternatives. *Structural Equation Modeling: A Multidisciplinary Journal*, *6*(1), 1–55. doi:10.1080/10705519909540118

[CIT0025] Keleş, S., & Yurt, Ö. (2017). An investigation of playfulness of pre-school children in Turkey. *Early Child Development and Care*, *187*(8), 1372–1387. doi:10.1080/03004430.2016.1169531

[CIT0026] La Paro, K. M., Hamre, B. C., & Pianta, R. C. (2009). *The classroom assessment scoring system, toddler version*. Charlottesville: Teachstone.

[CIT0027] Lieberman, J. N. (1977). *Playfulness. Its relationship to imagination and creativity*. New York: Academic Press.

[CIT0028] Mashburn, A. J., Pianta, R. C., Hamre, B. K., Downer, J. T., Barbarin, O. A., Bryant, D., … Howes, C. (2008). Measures of classroom quality in prekindergarten and children’s development of academic, language, and social skills. *Child Development*, *79*(3), 732–749. doi:10.1111/j.1467-8624.2008.01154.x 18489424

[CIT0029] Muthén, L. K., & Muthén, B. O. (1998-2018). *MPlus user’s guide* (*8th ed.*). Los Angeles: Muthén & Muthén.

[CIT0030] Okimoto, A. M., Bundy, A., & Hanzlik, J. (2000). Playfulness in children with and without disability: Measurement and intervention. *The American Journal of Occupational Therapy*, *54*(1), 73–82. doi:10.5014/ajot.54.1.73 10686630

[CIT0031] Pakarinen, E., Lerkkanen, M. K., Poikkeus, A. M., Kiuru, N., Siekkinen, M., Rasku-Puttonen, H., & Nurmi, J. E. (2010). A validation of the classroom assessment scoring system in Finnish kindergartens. *Early Education & Development*, *21*(1), 95–124. doi:10.1080/10409280902858764

[CIT0032] Peisner-Feinberg, E. S., Burchinal, M. R., Clifford, R. M., Culkin, M. L., Howes, C., Kagan, S. L., & Yazejian, N. (2001). The relation of preschool child-care quality to children's cognitive and social developmental trajectories through second grade. *Child Development*, *72*(5), 1534–1553. doi:10.1111/1467-8624.00364 11699686

[CIT0033] Perren, S., Frei, D., & Herrmann, S. (2016). Pädagogische Qualität in frühkindlichen Bildungs-und Betreuungseinrichtungen in der Schweiz [Pedagogical quality in early childhood education and care facilities in Switzerland] *Frühe Bildung*, *5*(1), 3–12. doi:10.1026/2191-9186/a000242

[CIT0034] Phillips, D. A., & Lowenstein, A. E. (2011). Early care, education, and child development. *Annual Review of Psychology*, *62*, 483–500. doi:10.1146/annurev.psych.031809.130707 20822436

[CIT0035] Pianta, R. C., Hamre, B. K., & Nguyen, T. (2020). Measuring and improving quality in early care and education. *Early Childhood Research Quarterly*, *51*, 285–287. doi:10.1016/j.ecresq.2019.10.013

[CIT0036] Pianta, R. C., & Hofkens, T. (2023). Defining early education quality using CLASS-observed teacher-student interaction. *Frontiers in Psychology*, *14*. doi:10.3389/fpsyg.2023.1110419 PMC10376698 37519392

[CIT0037] Pianta, R. C., La Paro, K., & Hamre, B. K. (2008). *Classroom assessment scoring system (CLASS)*. Boston: Paul H. Brookes.

[CIT0038] Pinchover, S. (2017). The relation between teachers’ and children’s playfulness: A pilot study. *Frontiers in Psychology*, *8*. doi:10.3389/fpsyg.2017.02214 PMC5742179 29312069

[CIT0039] Pinchover, S., & Shulman, C. (2019). Behavioural problems and playfulness of young children with ASD: The moderating role of a teacher’s emotional availability. *Early Child Development and Care*, *189*(14), 2252–2264. doi:10.1080/03004430.2018.1447934

[CIT0040] Pinchover, S., Shulman, C., & Bundy, A. (2016). A comparison of playfulness of young children with and without autism spectrum disorder in interactions with their mothers and teachers. *Early Child Development and Care*, *186*(12), 1893–1906. doi:10.1080/03004430.2015.1136622

[CIT0041] Pinto, A. I., Cadima, J., Coelho, V., Bryant, D. M., Peixoto, C., Pessanha, M., … Barros, S. (2019). Quality of infant child care and early infant development in Portuguese childcare centers. *Early Childhood Research Quarterly*, *48*(3), 246–255. doi:10.1016/j.ecresq.2019.04.003

[CIT0042] Reed, C. N., Dunbar, S. B., & Bundy, A. C. (2000). The effects of an inclusive preschool experience on the playfulness of children with and without autism. *Physical & Occupational Therapy in Pediatrics*, *19*(3-4), 73–89. doi:10.1300/J006v19n03_07

[CIT0043] Rentzou, K. (2013). Greek preschool children's playful behaviour: Assessment and correlation with personal and family characteristics. *Early Child Development and Care*, *183*(11), 1733–1745. doi:10.1080/03004430.2012.752736

[CIT0044] Rigby, P., & Gaik, S. (2007). Stability of playfulness across environmental settings: A pilot study. *Physical & Occupational Therapy in Pediatrics*, *27*(1), 27–43. doi:10.1300/J006v27n01_03 17298939

[CIT0045] Ruckser-Scherb, R. (2010). Die Beziehung von Spielfähigkeit und Effektivität des coping bei Kindergartenkindern im Alter von 4—6 Jahren [The relationship between playfulness and coping in preschool children 4–6 years of age]. *Ergoscience* *5*, 91–98. doi:10.1055/s-0029-1245547

[CIT0046] Salminen, J., Guedes, C., Lerkkanen, M. K., Pakarinen, E., & Cadima, J. (2021). Teacher–child interaction quality and children's self-regulation in toddler classrooms in Finland and Portugal. *Infant and Child Development*, *30*(3), e2222. doi:10.1002/icd.2222

[CIT0047] Salminen, J., Pakarinen, E., Poikkeus, A. M., Laakso, M. L., & Lerkkanen, M. K. (2022). Teacher-child interactions as a context for developing social competence in toddler classrooms. *Journal of Early Childhood Education Research*, *11*(1), 38–67. https://journal.fi/jecer/article/view/114006.

[CIT0048] Smidt, W., & Embacher, E. M. (2023). Examining the factorial validity of the individualized classroom assessment scoring system in preschools in Austria. *International Journal of Early Years Education*, *31*(3), 675–687. doi:10.1080/09669760.2021.1893158

[CIT0049] Thomason, A. C., & La Paro, K. M. (2009). Measuring the quality of teacher–child interactions in toddler child care. *Early Education and Development*, *20*(2), 285–304. doi:10.1080/10409280902773351

[CIT0050] Trevlas, E., Matsouka, O., & Zachopoulou, E. (2003). Relationship between playfulness and motor creativity in preschool children. *Early Child Development and Care*, *173*(5), 535–543. doi:10.1080/0300443032000070482

[CIT0051] Waldman-Levi, A., Bundy, A., & Shai, D. (2022). Cognition mediates playfulness development in early childhood: A longitudinal study of typically developing children. *The American Journal of Occupational Therapy*, *76*(5), 7605205020. doi:10.5014/ajot.2022.049120 35947034

[CIT0052] Whitebread, D., Neale, D., Jensen, H., Liu, C., Solis, S. L., Hopkins, E., … Zosh, J. M. (2017). *The role of play in children’s development: A review of the evidence*. Billund: The LEGO Foundation.

[CIT0053] Wood, E. (2007). New directions in play: Consensus or collision?. *Education 3-13*, *35*(4), 309–320. doi:10.1080/03004270701602426

[CIT0054] Wood, E., and Bennett, N. (1998). Teachers' theories of play: Constructivist or social constructivist?*.* *Early Child Development and Care*, *140*, 17–30. doi:10.1080/0300443981400103

[CIT0055] Wright, S. A. (2015). Playfulness in preschool children: A preliminary investigation into the impact of parental characteristics and child abuse and neglect (Doctoral dissertation). University of South Australia.

[CIT0056] Wustmann Seiler, C., Lannen, P., Duss, I., and Sticca, F. (2021). Mitspielen, (An)Leiten, Unbeteiligt sein?: Zusammenhänge kindlicher und elterlicher Playfulness: Eine Pilotstudie [Playing along, leading, being uninvolved? Parental play guidance and children’s playfulness]. *Frühe Bildung*, *10*(3), 161–168. doi:10.1026/2191-9186/a000526

[CIT0057] Youell, B. (2008). The importance of play and playfulness. *European Journal of Psychotherapy & Counselling*, *10*(2), 121–129. doi:10.1080/13642530802076193

